# A Study on the Heating and Deicing Performance of Microwave-Absorbing Asphalt Mixtures

**DOI:** 10.3390/ma16031051

**Published:** 2023-01-25

**Authors:** Yuyuan Deng, Xuancang Wang, Lv Chen, Mingyan Liu, Maohong Gao, Jing Zhao

**Affiliations:** School of Highway, Chang’an University, Xi’an 710064, China

**Keywords:** microwave deicing, magnetite powder, Fe_3_O_4_ powder, microwave heating, deicing performance

## Abstract

Road icing in winter brings challenges to traffic safety, and microwave heating and deicing technology is an effective method with the advantages of high efficiency and environmental protection. Magnetite has been widely used as a microwave-absorbing material in pavement. In this paper, magnetite powder formed by crushing natural magnetite and high-purity Fe_3_O_4_ powder after purification were mixed to replace mineral powder, and the magnetite aggregate was used to replace the limestone aggregate with the same particle size to enhance the asphalt mixtures’ microwave absorption capacity. The effect of microwave heating time and microwave power on the heating of the asphalt mixtures was studied, and the heating performance of different thicknesses of the asphalt mixtures under microwave radiation was evaluated. The heating performance of the mixtures under different initial temperatures and ice layer thicknesses was also assessed. The results showed that the addition of the magnetite powder–Fe_3_O_4_ powder and the magnetite aggregate significantly enhanced the heating performance of the asphalt mixtures by microwave heating. The replacement of the magnetite powder–Fe_3_O_4_ powder, the microwave heating time, and the microwave power had positive effects on the heating efficiency of the asphalt mixtures. Moreover, the thinner asphalt mixtures had a better heating performance. The heating and deicing performance of the mixtures decreased with a decline in initial temperature. As the ice thickness increased, the deicing time of the specimen surface to reach 0 °C also increased.

## 1. Introduction

In severe winter weather, roads are susceptible to the formation of hard ice, which is difficult to remove and can potentially cause traffic accidents [1,2]. The traditional measures of ice melting include the manual method, the mechanical method, and the chemical snow-melting method [3]. Manually removing snow and ice is a time-consuming and low-efficiency method, which is currently assisted by large machines to remove snow on special road sections. The mechanical method involves forceful removal using special machinery that acts directly on the pavement ice layer [4]. However, it still has many problems, such as low removal rate, low efficiency for thick ice layers, and damage to the pavement [5]. The chemical deicing method, according to the specific chemical composition, is mainly classified into chlorine salts, organic agents, and mixed categories. However, the chemical method leads to corrosion on the road surface and concrete structures, causing pollution to plants, soil, and water [6,7]. Fay et al. [8] summarized the possible risks of using chloride salts as freezing-point depressants. The excessive use of NaCl will increase the cation Na^+^ and anion Cl^−^. If these ions penetrate into groundwater, they will inevitably affect aquatic systems, and if they penetrate into soil, they will increase the salt content of the soil, which will also affect human health. Consequently, considering the drawbacks of traditional deicing methods, it is imperative to develop green, environmentally friendly, and efficient technology for road deicing and snow removal.

Microwave heating is a relatively well-developed industrial technology with a history of about 80 years of application in industrial heating, industrial packaging, medical treatment, food processing, and household appliances [9]. When considering the current status of microwave technology development, it is gradually being used in the road engineering of high-grade pavements, airport road surface ice melting, and maintenance management [10,11]. A road under microwave radiation converts microwave energy into thermal energy, which heats up the pavement and reduces the adhesion between the road and the ice layer, leading to the easy removal of the ice layer. The energy conversion and the deicing effect are even more obvious on roads containing microwave-absorbing materials [12]. Microwave heating and deicing technology has environmental and energy-saving advantages, overcomes the shortcomings of mechanical deicing that relies on a substantial amount of human and material resources, and has significantly higher efficiency than the mechanical method. It has promising potential for the intelligent automatic maintenance of deicing technology [13].

In pavement engineering, considerable studies have been carried out on deicing technology in terms of microwave-absorbing materials, construction process, and pavement structure. Hopstock et al. [14,15] first blended taconite material into an asphalt mixture to increase the electromagnetic properties of the mixture and successfully applied it to a demonstration project. Some researchers used waste materials to increase the performance of asphalt concrete. Suksiripattanapong et al. [16] used a high-density polyethylene (HDPE) plastic waste as an additive to improve the performance of an asphalt concrete pavement. It was found that when the content of the HDPE plastic waste was 5% of the aggregate weight, the HDPE plastic waste sample obtained the maximum resilient modulus, indirect tensile fatigue, and pavement age. Wang et al. [17] used steel slag from industrial waste to replace basaltic aggregates to improve the heating properties of asphalt mixtures. Liu et al. [18] modified steel slag particles using the coprecipitation method and evaluated the microwave absorption efficiency as a function of electromagnetic properties and microwave heating rate. Gao et al. [19] also used steel slag instead of aggregates and obtained specimens with an ice-melting efficiency about 2.6 and 2.3 times higher than the ordinary mixture at −5 °C and −20 °C, respectively. Furthermore, Gao et al. [20] investigated the microwave absorption efficiency of asphalt mixtures containing steel wool fibers and analyzed the effect of the initial temperature and the thickness of the ice layer on the specimens. Although using steel slag as an aggregate in asphalt mixtures has obtained favorable results, the bonding of the steel slag with the asphalt and asphalt aging need to be further discussed.

Micheli et al. [21] found that blending carbon nanotubes in concrete could enhance its electromagnetic shielding effectiveness. Tiyasangthong et al. [22] evaluated the possibility of using high-calcium fly ash geopolymers to stabilize a recycled concrete aggregate. Huang et al. [23] studied the deicing efficiency of concrete surfaces with added carbon fibers under multiple factors and obtained an optimum fiber length of 0.6 cm, while the initial temperature and the ice layer had little effect on microwave heating. Wang et al. [24] blended magnetite and carbon fiber as the microwave absorbers and showed that the deicing efficiency of the asphalt pavement was enhanced to some extent. Jahanbakhsh et al. [25] investigated the effects of adding carbon black to an asphalt concrete to enhance its electromagnetic sensitivity. The results showed that the mechanical properties of the microwave-absorbing asphalt concrete were excellent after adding the carbon black. Liu et al. [26] designed an asphalt concrete with a SiC–Fe_3_O_4_ microwave functional layer and showed that the melting time was 6.2 times lower than the control group. Although using fibers as microwave-absorbing materials has a strong ability to absorb heat, their cost is relatively high, and the dispersion uniformity in the asphalt mixture is hard to guarantee.

Similarly, using carbon black and a functional layer as the absorbing component of the pavement has the problem of high construction cost. Gulisano et al. [27] investigated the difference in dielectric properties between an asphalt mastic and an electric arc furnace slag mastic. The results showed that adding an electric arc furnace enhanced the dielectric properties of the asphalt mastics. Fernández et al. [28] analyzed long-term aging on the rheology of asphalt using frequency scanning tests and Fourier-transform infrared spectroscopy. Their study confirmed that microwave heating would not significantly affect the rheology of asphalt. Zhou et al. [29] investigated the relationship between FeSiAl/Al_2_O_3_ content and microwave reflection loss using the complex dielectric constant test. Chen et al. [30] studied road surface temperature under different microwave source heights and ice layers. Liu et al. [31] used activated carbon powder to replace mineral powder to enhance the deicing capacity and showed that the microwave energy conversion rate and the deicing capacity increased as the amount of activated carbon powder substitution increased.

In recent years, there has also been a series of research on using magnetite, magnetite powder, and Fe_3_O_4_ powder in microwave deicing pavements. Giustozzi et al. [32] used a rheology analysis to investigate the effect of adding magnetite to asphalt pavements and obtained the potential value of the magnetite filler with improved rheological properties, indicating that magnetite was a valuable road material. Al-Kheetan et al. [33] used 50% magnetite coarse aggregate to replace granite and gravel and showed that the SMA mixture with magnetite and granite had the maximum stiffness after aging, while the mixture containing magnetite and gravel had more slip resistance. Wang et al. [34] used sintering methods to prepare magnetite aggregates and mortars and evaluated the effects of magnetite content and ambient temperature on the deicing performance. Wang et al. [35] also used magnetite aggregates as microwave-absorbing materials and found that the heating efficiency of the magnetite aggregates was 5.15 times higher than that of the basaltic aggregates. Guo et al. [36] studied the heating capacity and radiation depth of magnetite aggregates under the action of microwaves, showing the potential ability of the magnetite aggregates to deice pavements in frozen areas. Yoshikawa et al. [37] observed that Fe_3_O_4_ powder was heated rapidly under a 2.45 GHz microwave field, indicating that Fe_3_O_4_ was an ideal material for microwave heating and had excellent microwave-absorbing properties. Ding et al. [12] found that a 1% mass ratio of Al_3_O_4_ and Fe_3_O_4_ mixed 1:1 at 2.45 GHz had a better deicing efficiency. Guan et al. [38] studied the microwave heating performance of adding magnetite powder to an asphalt mixture and found that increasing the dosage of magnetite powder increased the heating rate and the low-temperature performance. The recommended dosage of magnetite powder was 60%.

The existing studies on magnetite as a pavement microwave-absorbing material mainly focused on microwave heating experiments by replacing the aggregates in asphalt mixtures with magnetite. However, there have been few studies on mixing different microwave-absorbing fillers to replace mineral powder to enhance the deicing efficiency of asphalt mixtures. In this study, different volume percentages of magnetite powder and Fe_3_O_4_ powder were mixed to replace limestone powder. The magnetite aggregate was used to replace 2.36–4.75 mm of the limestone aggregate with the same particle size to prepare the microwave-absorbing asphalt mixtures. The average surface temperature of the specimen was measured by using a microwave oven and an infrared thermal imager, and the ice-melting time was recorded. The effects of the microwave heating time, the microwave power, the microwave-absorbing mixture thickness, the initial temperature, and the ice layer thickness were evaluated. This study provides a new idea for the use of mixed microwave-absorbing materials in pavement deicing technology. The main objectives of this study were as follows: (1) to evaluate the heating performance of the microwave-absorbing asphalt mixtures under microwave; (2) to recommend a reasonable thickness of the microwave-absorbing asphalt mixtures; and (3) to investigate the effect of the deicing efficiency of the microwave-absorbing asphalt mixtures under different working conditions.

## 2. Materials and Methods

### 2.1. Raw Materials

#### 2.1.1. Asphalt

In this study, asphalt (90#, Karamay Petrochemical Company, Karamay, China) was used as a binder. Table 1 shows the physical properties of the binder, which conform to the Standard Test Methods of Bitumen and Bituminous Mixtures for Highway Engineering in China (JTG E20-2011, 2011) [39,40].

#### 2.1.2. Aggregates

**Figure 1:**
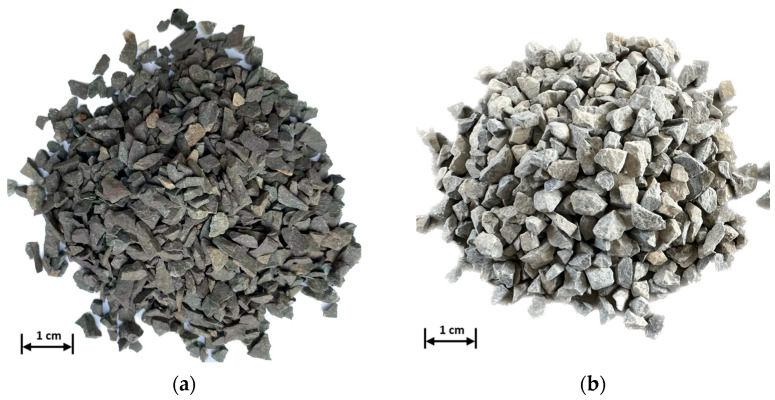
Aggregates: (**a**) Magnetite and (**b**) limestone.

Magnetite and limestone were used as the aggregates for this test. The magnetite was produced by Lingshou Huatai Minerals Co., Ltd., Shijiazhuang, China, while the limestone aggregates were from Chicheng Trading Co., Ltd., Xi’an, China; the aggregates are shown in Figure 1. The property tests of both aggregates were carried out according to the Test Methods of Aggregate for Highway Engineering (JTG E42-2005) [41]. The test results are shown in Table 2.

#### 2.1.3. Fillers

1. Fe_3_O_4_ powder

Three fillers were used in this study to prepare the asphalt mastic: Fe_3_O_4_ powder, magnetite powder, and limestone powder, which is commonly used in asphalt mixtures as the control group. The magnetite powder used in this paper was directly crushed from a natural magnetite ore, while the Fe_3_O_4_ powder used was a high-purity powder of magnetite after ore purification, and its purity reached 99%. There were differences in the preparation process and Fe content. The physical properties of the Fe_3_O_4_ powder, the magnetite powder, and the limestone powder were tested according to the Test Methods of Aggregate for Highway Engineering (JTG E42-2005) [41]. The test results are shown in Table 3.

**Figure 2:**
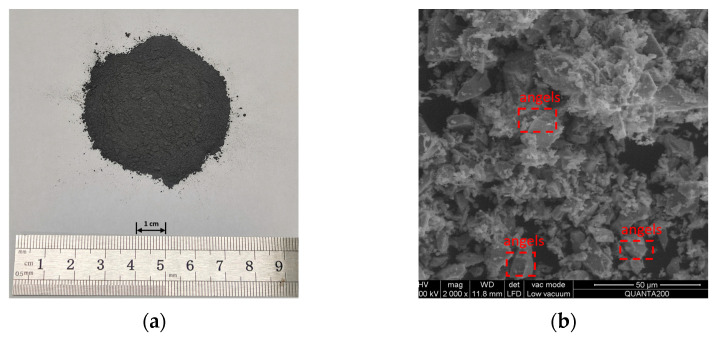
Fe_3_O_4_ powder: (**a**) appearance, and (**b**) ESEM image of the Fe_3_O_4_ powder.

**Figure 3:**
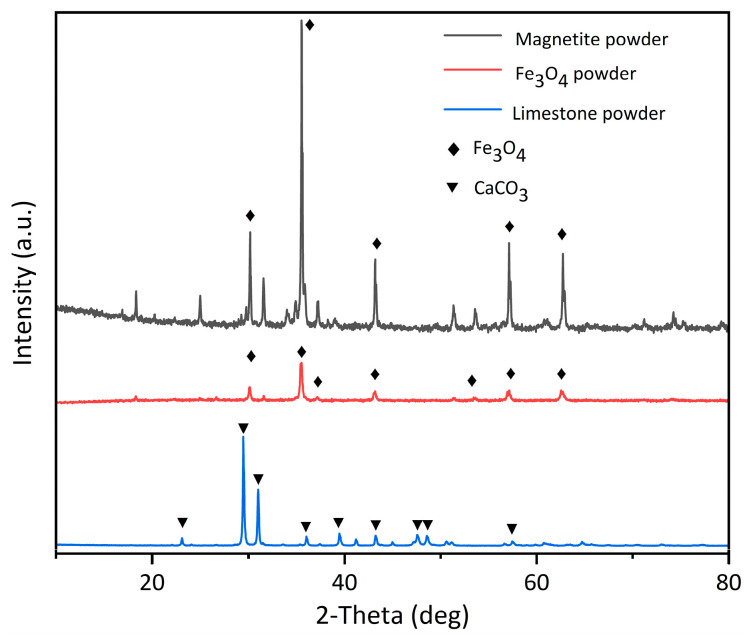
XRD of the Fe_3_O_4_ powder, the magnetite powder, and the limestone powder.

The high-purity Fe_3_O_4_ powder was produced by China Metallurgical Research Institute. Its microstructure was observed using an environmental scanning electron microscopy (ESEM) with 2000× magnification; as shown in Figure 2b, the microstructure of the Fe_3_O_4_ powder is mainly blocky particles with obvious angularity, a small particle size, a uniform particle size distribution, a large surface area, and better microscopic roughness, indicating that the Fe_3_O_4_ powder had better adhesion with the asphalt after mixing. The X-ray diffraction (Panalytical Empyrean, Almelo, The Netherlands) reveals the mineral composition of the aggregates, where the scanning range is from 10° to 90° at a rate of 2°/min, as shown in Figure 3.

Related studies showed that the Fe_3_O_4_ powder in a humid condition would react with oxygen, thus reducing its heating performance; therefore, the Fe_3_O_4_ powder needed to be dried to obtain a better microwave heating performance [37]. Accordingly, the Fe_3_O_4_ powder in this study was treated in a temperature oven at 150 °C for 12 h before use.

2. Magnetite powder

**Figure 4:**
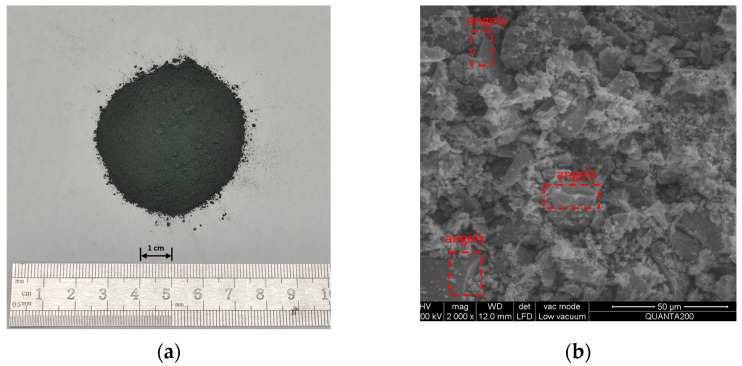
Magnetite powder: (**a**) appearance, and (**b**) ESEM image of the magnetite powder.

The magnetite powder was obtained from Lingshou Huatai Minerals Co., Ltd., Shijiazhuang, China, and its physical properties are shown in Table 3. In Figure 4b, its microscopic characteristics are observed using an environmental scanning electron microscopy (ESEM) with 2000× magnification. The magnetite powder has obvious angular and tabular shapes, as well as a crystalline structure, which is beneficial to crosslinking with asphalt. Fe_3_O_4_ is the main component of the magnetite powder, as observed in the X-ray diffraction in Figure 3. The magnetic component gives the hybrid material better magnetic properties and improves its microwave absorption performance.

3. Mineral powder

**Figure 5:**
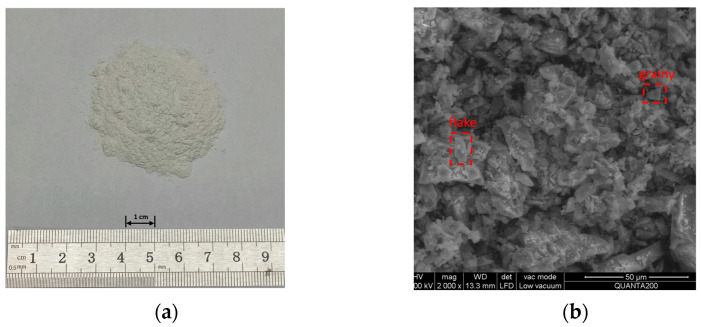
Mineral powder: (**a**) appearance, and (**b**) ESEM image of the mineral powder.

The limestone mineral powder was obtained from Chicheng Trading Co., Ltd., Xi’an, China. Its physical properties are shown in Table 3. The appearance and microscopic shape are shown in Figure 5. It can be found that the limestone mineral powder is mostly blocky in large particles and flaky and grainy in small particles. As can be seen from the XRD in Figure 3, CaCO_3_ is the main component of the limestone mineral powder.

### 2.2. Sample Preparation

#### 2.2.1. Preparation of Asphalt Mastic

The fillers used in this study included the limestone mineral powder, the magnetite powder, and the Fe_3_O_4_ powder. In 2022, the wholesale price of Fe_3_O_4_ powder with an average particle size of 50 µm in Shijiazhuang, China, was about 2500 RMB/t, while natural magnetite powder was about 700 RMB/t. In a previous microwave-heating efficiency test of asphalt mastic, it was found that the heating performance of the asphalt mastic blended with the Fe_3_O_4_ powder was higher than that of the magnetite powder under microwave heating. It is noteworthy that the magnetite powder with a volume ratio of 80% and the Fe_3_O_4_ powder with a volume ratio of 20% were mixed and recorded as CFe. It was found that the average heating rate of the asphalt mastic prepared by mixing the magnetite and Fe_3_O_4_ powders was up to 80% of that of the asphalt mastic prepared by the Fe_3_O_4_ powder. With this replacement ratio, the filler cost was reduced by approximately 58%. Therefore, the magnetite powder was chosen as the main filler for the preparation of the microwave-absorbing asphalt mastic. In this paper, the CFe was used instead of the limestone powder to prepare a mixed microwave-absorbing mastic with both microwave-absorbing heating performance and economic characteristics.

**Figure 6:**
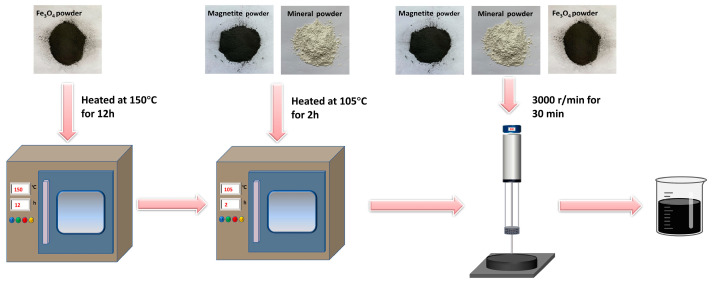
Flowchart of asphalt mastic preparation.

In this study, the magnetite powder, mineral powder, and oven-treated Fe_3_O_4_ powder were put into different test beakers and then into the oven at 105 °C for 2 h until the quality of the filler did not change; an appropriate quantity of asphalt was put into the oven with the temperature at 150 °C for 1 h. The mass ratio of the mixed filler to asphalt was chosen as 0.36:1. First, 108 g of the mixed filler was weighed and gradually blended into 300 g of heated asphalt at high speed (3000 r/min) in a high-speed stirrer for 30 min. The CFe was used to replace the limestone mineral powder at different volume fractions (0%, 25%, 50%, 75%, and 100%) to prepare five microwave-absorbing asphalt mastics. The sample preparation process is shown in Figure 6.

#### 2.2.2. Preparation of Asphalt Mixture

**Figure 7:**
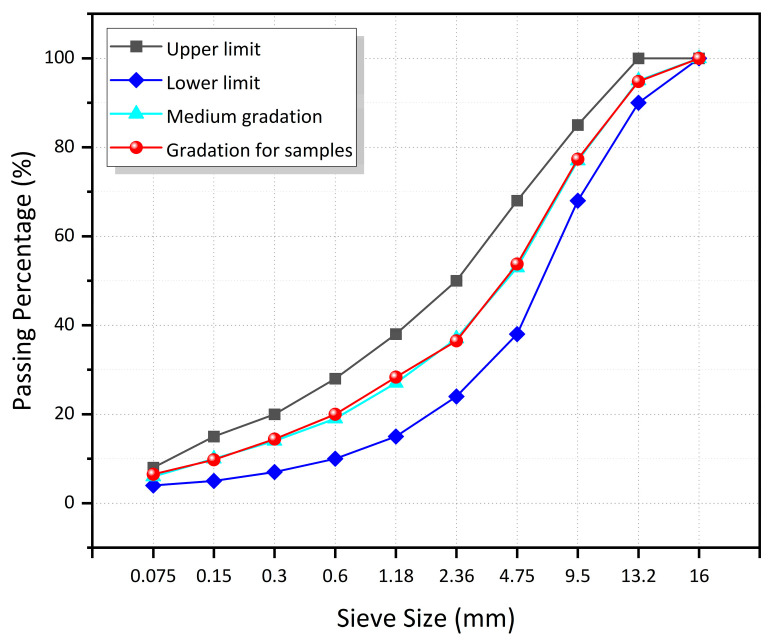
AC-13 gradation of the aggregate.

It is well known that large-particle-size aggregates often carry most of the traffic loads in asphalt mixtures [42]. Therefore, to avoid changing the skeleton structure, the magnetite aggregates were used to replace all limestone aggregates of small particle size (2.36 mm to 4.75 mm) by equal volume. This paper uses the dense-graded asphalt concrete with AC-13 for grade design, which is commonly used for highway pavement structures in China [40], and the grading curve is shown in Figure 7. According to the CFe from 0 to 100 vol.% (increment of 25 vol.%) of the five replacement grades to replace the limestone mineral powder as filler, the magnetite aggregate was used to replace the limestone aggregate of the same particle size to prepare the microwave-absorbing asphalt mixtures, and the corresponding samples were named CFe0, CFe25, CFe50, CFe75, and CFe100, along with a control group of ordinary asphalt mixture. The optimum asphalt–aggregate ratio of the asphalt mixture was determined using the Marshall test method conforming to the Standard Test Methods of Bitumen and Bituminous Mixtures for Highway Engineering in China (JTG E20-2011, 2011) [40]. The test scheme and the optimum asphalt dosage of the Marshall specimens of asphalt mixtures are shown in Table 4.

### 2.3. Test Methods

#### 2.3.1. Microwave Heating Test

**Figure 8:**
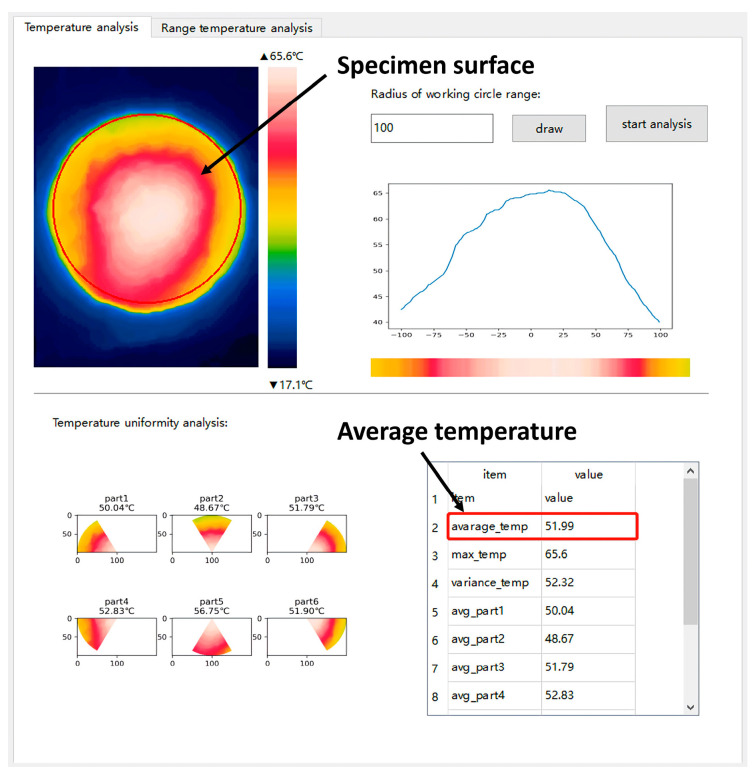
Surface temperature test of the specimens.

Microwave heating time tests were conducted on the microwave-absorbing asphalt mixtures and the ordinary asphalt mixture, and the specimens were heated under the microwave. The Marshall specimens were prepared in accordance with the Chinese specifications (JTG E20-2011) [40]. One control group and five absorbing Marshall specimens with CFe were prepared to study heating performance under microwave heating. Accordingly, parallel specimens were set in each group, for a total of 18 Marshall specimens. Then, the prepared specimens were controlled at a room temperature of 20 °C (±0.5 °C). The microwave heating equipment for the asphalt mixtures was adopted from a high−power commercial microwave oven (MWO 38 IX, Whirlpool (China) Co., Ltd., Hefei, China) with 800 W power and a microwave frequency of 2.45 GHz. An infrared thermal imager (UTi120S, Uni-Trend Technology (China) Co., Ltd., Dongguan, China) was used to measure the average temperature on the surface of the Marshall specimens at 0, 2, 4, 6, 8, and 10 min, and the temperature was measured for each specimen after a 2 min heating cycle. The measurement time was controlled within 5 s. The temperature data of the infrared thermal imager were exported from the computer. The average temperature of the specimen surface was used as the measuring temperature. As shown in Figure 8, the test results are taken as the average values of the parallel specimens.

#### 2.3.2. Microwave Heating Power Test

Generally speaking, a higher input power of microwaves results in a considerably higher total amount of electromagnetic wave energy entering into the absorbing material and more heat being transformed. Therefore, the relationship between the microwave power and the heating performance of the microwave-absorbing asphalt mixtures was studied. To reduce the error caused by repeatedly opening the microwave oven, as well as the large errors caused by too-low heating time, the heating time of the specimens was set at 8 min. Four microwave power levels (700 W, 800 W, 900 W, and 1000 W) were set in this paper. Before the test, all Marshall specimens were thermostatically treated to ensure that the temperature of the specimens was 20 ± 0.5 °C. The surface temperature of the Marshall specimens was measured after heating for 8 min. The average temperature of the parallel specimens was used as the heating temperature.

#### 2.3.3. Microwave Heating Efficiency Test

The relationship between the surface temperature and the heating time of a microwave-absorbing asphalt mixture under microwave heating essentially reflects the effect of different replacement rates of the microwave-absorbing materials on the heating performance. To study the heating rate of the microwave-absorbing asphalt mixtures, one group of ordinary Marshall specimens was prepared as the control group, and five groups of the microwave-absorbing asphalt mixtures with different CFe replacement rates were also prepared for the microwave heating test. The heating rate K was calculated using Equation (1). The average heating rate K was calculated for different replacement rates of the microwave-absorbing asphalt mixtures in the periods of 0–2 min, 2–4 min, 4–6 min, 6–8 min, and 8–10 min, as well as in the whole 0–10 min period. To study the temperature increment per second of the absorbing asphalt mixtures, the difference in heating rate ΔK was calculated using Equation (2).

(1) $$K=\frac{T_{2}-T_{1}}{t_{2}-t_{1}},$$

(2) $$\Delta K=K_{2}-K_{1},$$

Here, $T_{2}$ is the cross-sectional temperature at $t_{2}$ (°C); $T_{1}$ is the cross-sectional temperature at $t_{1}$ (°C); $t_{2}-t_{1}$ is the time from $t_{1}$ to $t_{2}$ (s); and $K_{i}$ is the heating rate of the absorbing material under a certain condition (°C/s).

#### 2.3.4. Microwave-Absorbing Layer Thickness Test

It is significant to study the heating performance of different thicknesses of a microwave-absorbing mixture so as to determine a reasonable microwave-absorbing pavement structure. At present, the popular structure for highway pavements in China is a 4 cm upper layer + 6 cm middle layer + 8 cm lower layer [43]. According to the microwave oven parameters used (800 W and 2.45 GHz), the microwave wavelength at this frequency is 122 mm. Ding et al. [12] concluded that the relative dielectric constant of asphalt concrete is 4.5–6.5 and the loss angle tangent value is 0.015–0.036. According to Equation (3), the minimum value of microwave penetration depth is 21.18 cm. This value is greater than the asphalt mixture layer thickness of 18cm; thus, it can be seen that the entire thickness of the surface is within the microwave heating depth.

(3) $$D=\frac{\lambda_{0}}{2\Pi \tan\delta \sqrt{\varepsilon }},$$

Here, D is the microwave penetration depth (mm), $\lambda_{0}$ is the microwave wavelength, ε is the relative dielectric constant, and tanδ is the loss angle tangent value.

**Figure 9:**
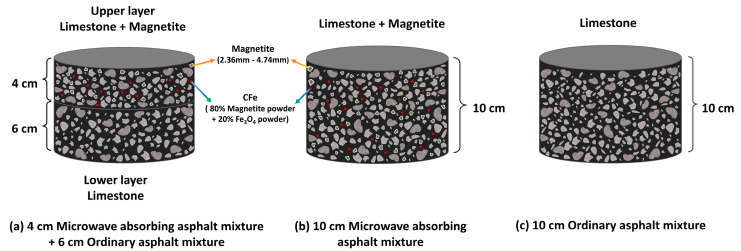
The schematic diagram for the Marshall specimens of the asphalt mixtures.

Therefore, according to the thicknesses of the upper and middle layers, two kinds of microwave-absorbing mixture specimens of 4 cm and 10 cm thickness were designed, including a 4 cm microwave-absorbing mixture as the upper layer + a 6 cm ordinary Marshall specimen as the lower layer, as well as a 10 cm microwave-absorbing asphalt mixture and a 10 cm ordinary asphalt mixture. In addition, the standard Marshall specimen (101.6 diameter × 63.5 mm) was used in the heating test for comparison with the CFe50 and CFe100 microwave-absorbing asphalt mixture specimens, according to the mixture design in Figure 9. The time was set to 10 min, and the temperature of the specimen surface was measured every 2 min.

#### 2.3.5. Surface Temperature Test of Marshall Specimens at Low Temperature

**Figure 10:**
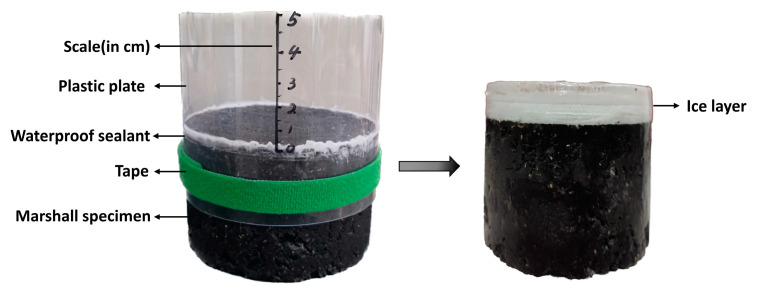
The preparation of the ice layer.

Ambient temperature has a substantial effect on the heating performance of microwave-absorbing asphalt mixtures. To investigate the effect of the initial temperature on the deicing efficiency of the microwave-absorbing asphalt mixtures, a plastic sheet was used to surround the perimeter of the Marshall specimen, which is waterproof and higher than the Marshall specimen. A waterproof sealant was used at the joint between the edge of the specimen and the plastic plate, and the plastic plate was tied with tape and marked with a scale. Water was added to the specimen surface to 1cm, and the specimen was placed into a temperature-controlled freezer at −5 °C, −10 °C, and −15 °C for 24 h. The preparation of the ice layer is shown in Figure 10. A microwave oven (800 W power, 2.45 GHz) was used to heat the CFe50 and CFe100 microwave-absorbing asphalt mixtures. To prevent the melted ice from boiling under the effect of the microwave, the heating time was set to 8 min; the temperature of the specimen surface was measured every 2 min, and the microwave oven was opened within 5 s. The time taken for each specimen to heat up to 0 °C was calculated by interpolating the temperature rise curve.

#### 2.3.6. Temperature Test of Marshall Specimen Surface Covered with Ice Layer

The efficiency of microwave deicing is influenced by the ice layer thickness in terms of the freezing and bonding of the ice layer to the asphalt surface [44]. To simulate the effect of different ice layer thicknesses on the deicing performance of the microwave-absorbing asphalt mixtures, three ice layer thicknesses of 1 cm, 3 cm, and 5 cm were used. The preparation of the ice layer is shown in Figure 10. The Marshall specimens were covered with different thicknesses of ice layer and placed in a thermostatic freezer at −5 °C for 24 h. Then, a microwave oven (800 W and 2.45 GHz) was used to heat the ice-covered specimens for a total heating time of 8 min, the temperature of the specimen surface was recorded every 2 min, and the time for measuring the temperature did not exceed 5 s.

## 3. Results and Discussion

### 3.1. Effects of Microwave Heating Time

**Figure 11:**
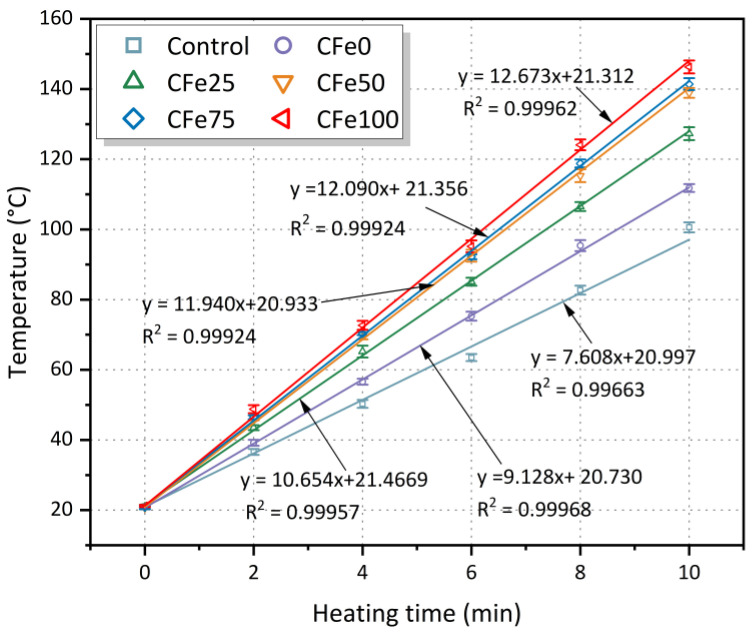
Surface temperature of the specimens under different microwave heating times.

Figure 11 shows the relationship between the heating time and the temperature of the specimens with magnetite aggregates and different amounts of CFe. As can be seen from Figure 11, the surface temperature of the asphalt mixture mixed with CFe is significantly higher than that of the control group, and the temperature increases with the replacement rate of CFe. This heating efficiency is similar to the results obtained by Guan et al. [38], who added magnetite powder as a microwave-absorbing material to asphalt concrete and found that the microwave heating rate of the asphalt mixture with magnetite powder was significantly higher than that of traditional asphalt mixture.

When the CFe replaces the limestone mineral powder at 100%, the surface temperature of the CFe100 increases from a room temperature of 20 °C to 146.3 °C under microwave heating for 10 min, which is 5 °C, 7.4 °C, 19 °C, 34.5 °C, and 45.7 °C higher than the increase recorded for the CFe75, the CFe50, the CFe25, the CFe0, and the control group, respectively (3.5%, 5.3%, 14.9%, 30.8%, and 45.4% increases, respectively). It can be seen that, when using magnetite powder and Fe_3_O_4_ powder in the mixture instead of limestone powder, a higher replacement rate of CFe leads to a higher surface temperature of the mixture, indicating a better heating performance. The surface temperature of the specimens with different CFe content is linearly related to the microwave heating time; R^2^ is more than 0.99, indicating that the temperature of the specimen surface and the heating time correlate well. It can also be seen from Figure 11 that for CFe0, which uses 2.36–4.75 mm of magnetite aggregate instead of limestone aggregate of the same particle size, the temperature of the specimen surface rises from 20 °C to 112 °C for 10 min, which is about 1.33 °C per minute more than the control group, indicating that the addition of both CFe and magnetite aggregate as the microwave-absorbing materials could enhance the heating capability of the asphalt mixtures by microwaves.

### 3.2. Effects of Microwave Power on Heating Performance

**Figure 12:**
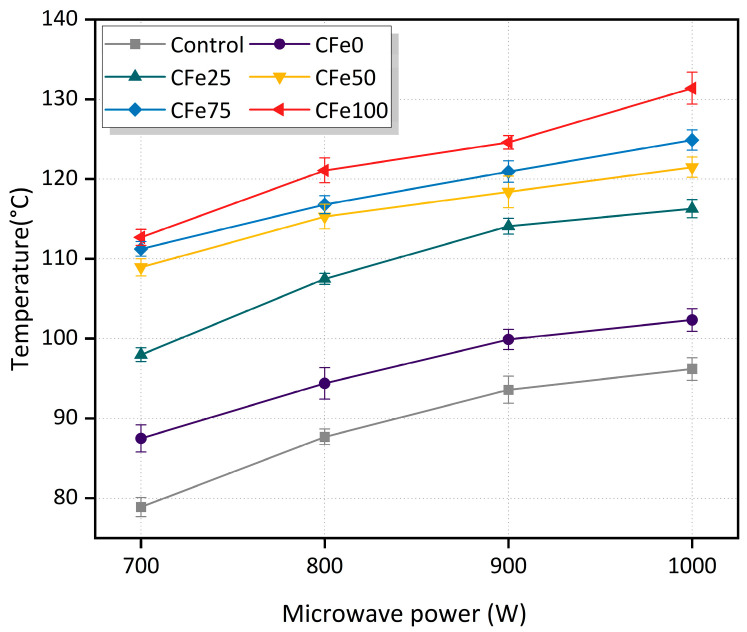
Change in the surface temperature of the specimens with microwave power at 8 min.

As can be seen from Figure 12, the increased microwave power improves the surface heating capacity of both the ordinary asphalt mixtures and the microwave-absorbing asphalt mixtures. In particular, as the microwave power increases from 700 W to 800 W, the rise in temperature on the specimen surface is higher than that observed from 800 W to 900 W and from 900 W to 1000 W, regardless of the CFe replacement rate. Moreover, the temperature of the specimen surface at 8 min increases with the CFe replacement rate for a certain microwave power.

Notably, the CFe25 specimen shows a maximum temperature difference of 18.3 °C as the power increases from 700 W to 1000 W, indicating that the heating performance of the CFe25 is more sensitive to microwave power. For the CFe50 and CFe75 specimens, the power increase has less influence on the heating performance, with a temperature rise difference of 12.6 °C and 13.6 °C, respectively. These substitution rates can achieve good heating performance while reducing power consumption. Considering the practical application of the microwave-absorbing asphalt mixtures, the CFe25’s heating performance is sensitive to the change in microwave power, and the microwave power needs to be increased to obtain greater heating performance, which increases energy consumption. Its heating capacity is not as good as the CFe mixtures with a replacement rate of ≥50%. To ensure a stable heating rate for the microwave-absorbing asphalt mixtures, a CFe replacement rate of ≥50% should be used in practice.

### 3.3. Microwave Heating Efficiency

**Figure 13:**
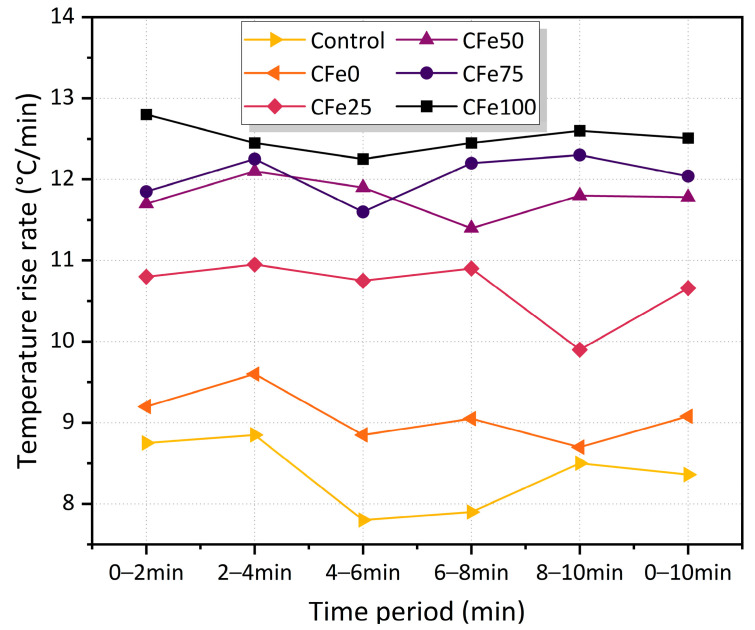
Change in microwave heating rate with heating time.

Figure 13 shows the heating rate of the specimens under microwave heating. It can be observed that the heating rate of the CFe100 is the maximum for all periods; its heating rate at 0–10 min is maintained at 12.5 °C/min, while the control group only reaches 7.9–8.7 °C/min and its rate fluctuates considerably. This may have been due to the initial temperature being set at 20 °C and the control group being heated up under microwave heating, while relying only on the asphalt and limestone aggregates. It is interesting to note that the heating rates of the surface temperatures of the CFe50 and the CFe75, as well as their heating performance, are roughly similar. The heating rate of the CFe25 from 0 to 10 min is about 10.6 °C/min, which is markedly higher than that of the CFe0 and the control group, but lower than that of the CFe50. Therefore, a replacement rate of CFe chosen to be ≥50% would obtain a better heating performance for the microwave-absorbing asphalt mixtures.

### 3.4. Effect of the Thickness of Microwave-Absorbing Layers on Heating Performance

**Figure 14:**
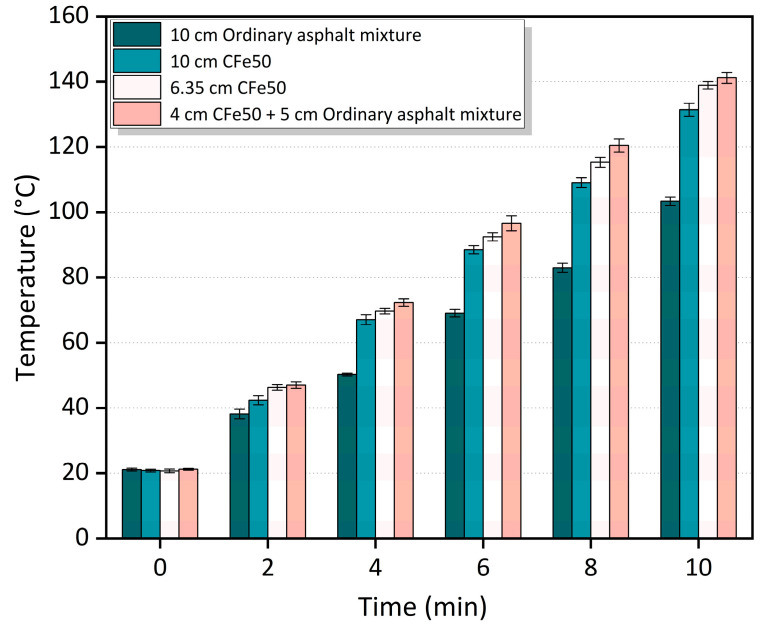
Temperature change for different thicknesses of the asphalt mixtures with a 50% replacement rate of CFe.

**Figure 15:**
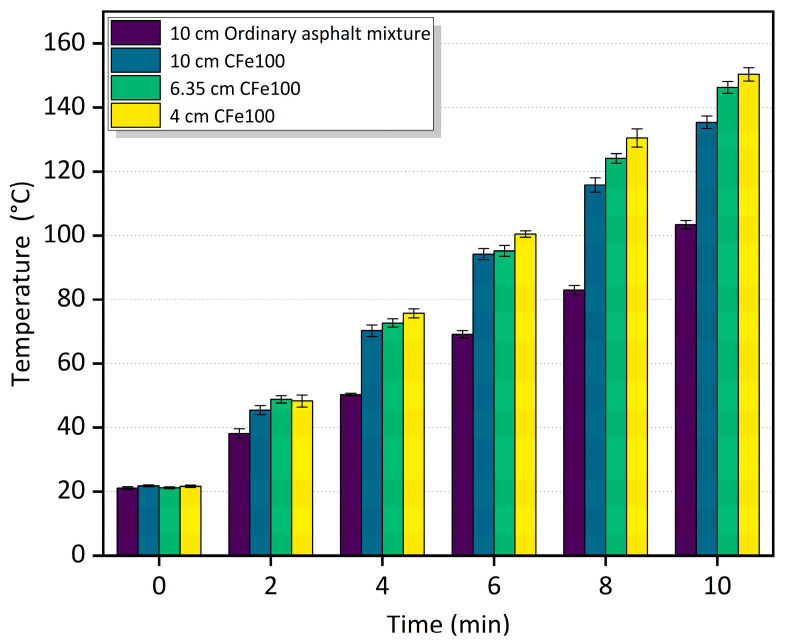
Temperature change for different thicknesses of the asphalt mixtures with a 100% replacement rate of CFe.

Figure 14 and Figure 15 show the relationship between the surface temperature of different thicknesses of the microwave-absorbing mixtures and time. In Figure 14, at a 50% CFe replacement rate, the average heating rate of the pavement structure of the 4 cm CFe50 + 6 cm ordinary asphalt mixture is 2.0%, 8.5%, and 45.8% higher than that of the 6.35 cm CFe50, the 10 cm Cfe50, and the 10 cm ordinary asphalt mixtures, respectively. It can be observed in Figure 15 that, at a 100% CFe replacement rate, the heating rate of the 4 cm CFe100 + 6 cm ordinary asphalt mixture is 2.9%, 13.3%, and 56.4% higher than that of the 6.35 cm CFe100, the 10 cm CFe100, and the 10 cm ordinary asphalt mixtures, respectively. It can be seen that the thickness of a microwave-absorbing mixture and the CFe replacement rate both significantly impact the heating performance of the mixture, with a lower thickness of the microwave-absorbing mixture showing a better heating performance. The main reason is that the upper layer of the asphalt pavement as a microwave-absorbing layer could maintain its heating performance at a high level, constantly generating and transferring heat to the asphalt surface and ice layer, which is conducive to improving the deicing efficiency. However, the heat generated by a thick microwave-absorbing mixture under microwave irradiation is dispersed in the middle layer and lower layer, thereby reducing the heating efficiency, heat conversion rate, and utilization rate of the microwave-absorbing mixture.

Therefore, combined with Section 3.1 and considering the typical pavement structure of highways and the heating performance of different microwave-absorbing layer thicknesses, it is recommended that only the upper layer of the 4 cm microwave-absorbing layer be used for microwave-absorbing highway asphalt pavements and that the middle and lower layers follow the common structure of highways.

### 3.5. Effect of Initial Temperature on Heating and Deicing Performance

**Figure 16:**
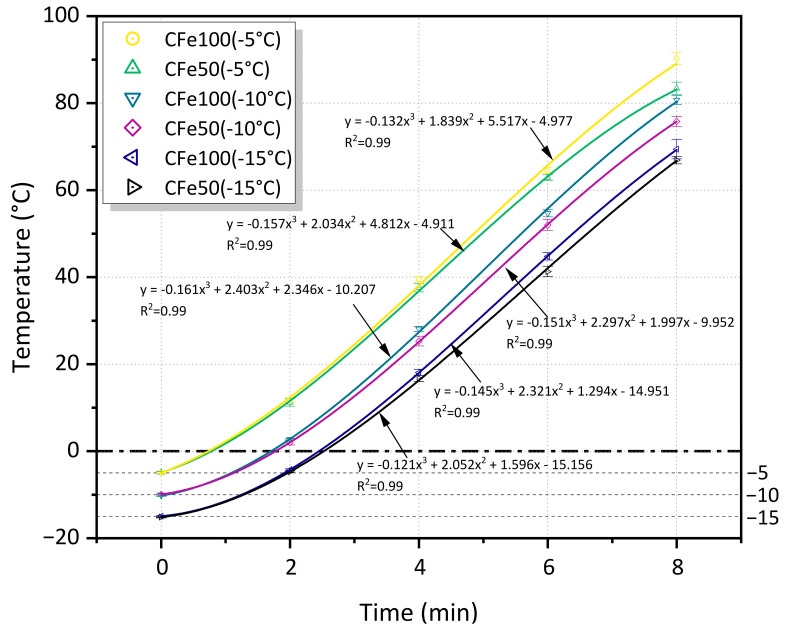
The surface temperature change of the specimens with microwave heating time under different initial ambient temperatures.

Figure 16 shows the heating performance of the microwave-absorbing asphalt mixture at the initial temperatures of −5 °C, −10 °C, and −15 °C. The difference in the surface temperature between the CFe50 and the CFe100 is not significant at the same initial temperature. The effect of the initial temperature on the heating performance of the microwave-absorbing asphalt mixtures is mainly observed when the temperature reaches 0 °C between the asphalt surface and the ice layer. Subsequently, the effect is lower, especially after the heating time reaches 2 min, whereby the heating rate of all groups reaches 11.3 to 12.4 °C/min (Figure 11).

In the practical deicing process, it is also sufficient for the temperature of the road surface to reach the deicing point of 0 °C with the microwave heating from a microwave vehicle. Therefore, the heating rate K and the heating time for the surface temperature to reach 0 °C within the first 2 min were calculated. As shown in Table 5, the heating rate of each specimen within the first 2 min shows a slight difference, with a maximum difference of only 0.32 °C/min. However, the heating rate within the first 2 min is substantially less than that after 2 min, with a difference in the heating rate between 4.8 and 6.7 °C/min. The CFe50 and the CFe100 surface temperatures take 151.4 s and 147.5 s to heat up from −15 °C to the 0 °C deicing point, which are 45 s and 44.8 s more than when starting at −10 °C, and 104.7 s and 103.7 s more than when starting at −5 °C. The results show that the initial temperature of the pavement surface has a significant impact on a microwave-absorbing mixture being heated to 0 °C. This is because a lower ambient temperature requires more electromagnetic losses to heat to overcome the temperature difference in the pavement structure and, thus, reach the deicing point at a longer time. The same trend was reported by Guo et al. [36]. They used magnetite instead of limestone aggregate and found that the deicing time of the asphalt mixture increased with a decrease in the initial temperature. The deicing time at the initial temperature of −20 °C was about 1.5 times that at −5 °C. Therefore, before a microwave-deicing vehicle operation, a low-temperature asphalt pavement section should be preheated at a low power for 1–2 min in advance, or the operation time should be extended to overcome the negative effect of the initial temperature factors on the deicing efficiency.

### 3.6. Effect of Ice Layer Thickness on Deicing Performance

**Figure 17:**
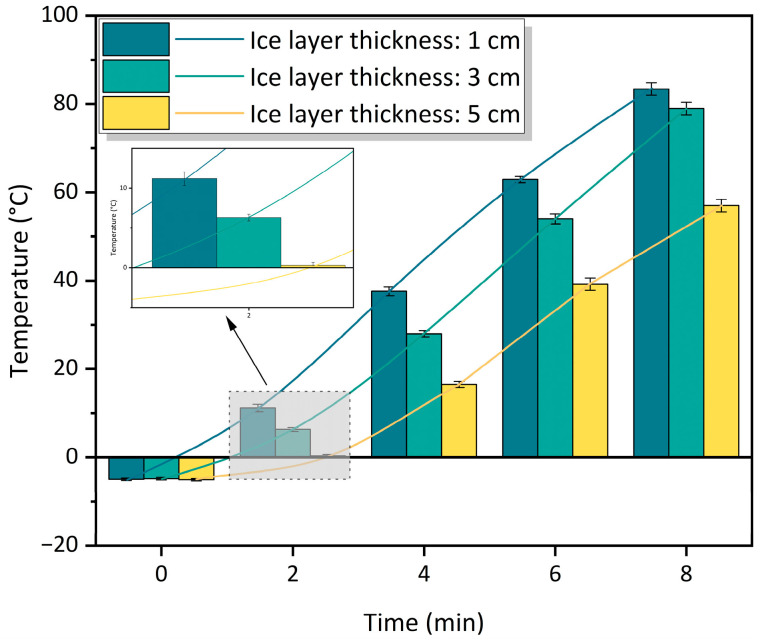
The temperature change in the specimen surfaces covered with different ice layer thicknesses.

Figure 17 shows the relationship between the temperature of the CFe50 surface covered with 1 cm, 3 cm, and 5 cm ice layers at the same initial temperature of −5 °C. The results show that the heating rate of the specimen surface decreases as the thickness of the ice layer increases. The temperature of the specimen surface covered with an ice layer of 1 cm increases from the initial temperature of −5 °C to 83.4 °C within 8 min, with an average heating rate of 10.42 °C/min, while the heating rates of the ice layer thickness of 3 cm and 5 cm are 9.7 °C/min and 7.1 °C/min, respectively. However, the practical microwave ice-melting process using a deicing truck only needs the temperature of the road surface to reach 0 °C. In Figure 17, the temperature of the specimen surface reaches above 0 °C within 3 min of heating, and the ice-melting times of the surface temperature to reach 0 °C are calculated to be about 38 s, 54 s, and 88 s according to the fitted curve. Gao et al. [20] also studied such conditions. They investigated the relationship between the deicing efficiency and the thickness of the ice layer cover on the road surface and found that an increase in the thickness of the ice layer increased the time of deicing, but the thickness of the ice layer had a limited effect on the deicing efficiency.

**Figure 18:**
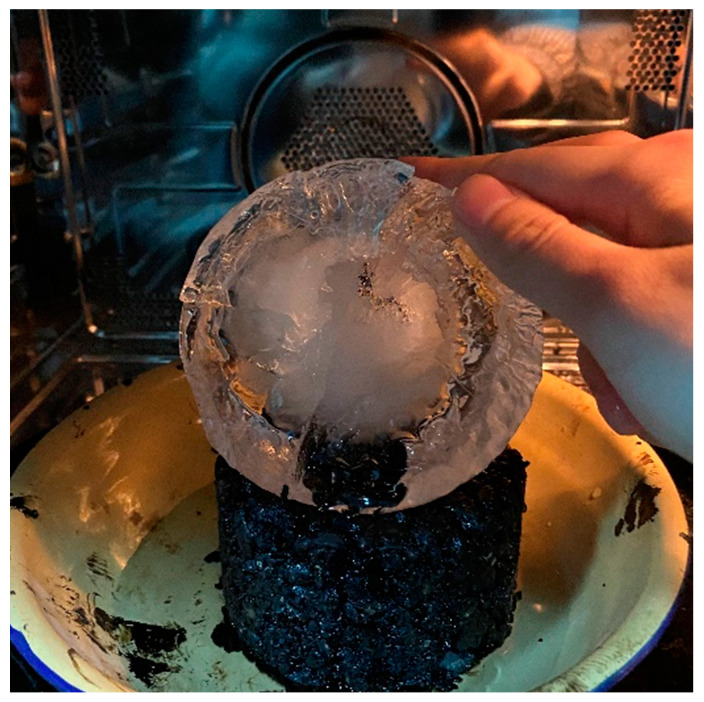
Inverted bowl-shaped ice layer after microwave heating.

In addition, it is noteworthy that the ice layer after microwave heating shows an inverted bowl shape (see Figure 18). The center of the ice layer in contact with the specimen surface is melted more, while the periphery is less. This is due to the microwaves radiating to the edge of the surface of the specimen being more reflected in the surroundings, while the microwave-absorbing materials in the mixture generate part of the heat emitted in the air, forming a shape where the thickness of the ice layer edge is greater than that in the center.

## 4. Conclusions

In this study, the heating and deicing performance of microwave-absorbing asphalt mixtures with different replacement rates of CFe were studied using a mixture of magnetite powder and Fe_3_O_4_ powder instead of limestone powder, while magnetite aggregate, instead of limestone aggregate with the same particle size, was used as the microwave absorber. Some conclusions are as follows:

(1) An increase in the replacement rate of CFe, heating time, and microwave power improved the heating performance of the mixture. To ensure a higher heating rate and stability of the microwave-absorbing asphalt mixture under microwave heating, the recommended CFe replacement rate is ≥50%.
(2) At a certain replacement rate of CFe, the thickness of the microwave-absorbing asphalt mixture had a negative impact on the heating performance. At 50% and 100% replacement rates of CFe, the heating rates of the 4 cm CFe50 and the CFe100 were 2.0% and 2.9% higher than that of the control group and 8.5% and 13.7% higher than that of the 10 cm layer, respectively. Therefore, a 4 cm upper layer is sufficient as a microwave-absorbing layer for pavement structures.
(3) A decrease in ambient temperature led to a reduction in deicing efficiency. The heating times for the CFe50 and the CFe100 from −15 °C to 0 °C were 151.4 s and 147.5 s, which were 1.42 and 1.44 times longer than those from −10 °C and 3.24 and 3.37 times longer than those from −5 °C.
(4) The deicing time increased with the thickness of the ice layer. The heating times of the CFe50 covered with an ice layer of 1 cm, 3 cm, and 5 cm to reach a surface temperature of 0 °C were approximately 38 s, 54 s, and 88 s, respectively.

This study has revealed that magnetite powder and Fe_3_O_4_ powder used as microwave absorbers can improve the deicing and heating performance of asphalt mixtures. The results obtained are favorable, but there are still some problems that need further study. Firstly, the effect of the magnetite from different sources and different grades of asphalt on the microwave-absorbing mixtures needs to be further determined. In addition, the fatigue durability properties of the microwave-absorbing materials in the asphalt mixtures can also be studied in detail. Finally, the mechanical properties of the microwave-absorbing asphalt mixtures at low-temperature crack resistance, water stability, and so on also need to be fully studied.

## Figures and Tables

**Table 1:** Properties of the asphalt binder.

Index	Unit	Test Results	Specification	Test Methods
Penetration (25 °C, 100 g, 5 s)	0.1 mm	89.2	80~100	T0604
Softening Point (TR&B)	°C	54	≥44	T0606
Ductility (15 °C, 5 cm/min)	cm	≥100	≥100	T0605
Flash point	°C	279	≥245	T0611
Density (15 °C)	g/cm^3^	1.009	N/A	T0603
Rolling Thin Film Oven Test (RTFOT)				
Loss of quality	%	0.36	≤±0.8	T0610
Penetration ratio (25 °C)	%	65	≥57	T0604
Ductility	cm	38.5	≥20	T0605

**Table 2:** Basic physical performance of the aggregates.

Aggregate Type	Crushing Value (%)	Los Angeles Abrasion Value (%)	Apparent Relative Density	Water Absorption (%)	Adhesion with Asphalt (≥)
Magnetite	9.6	9.2	3.814	0.45	5
Limestone	15.9	18.9	2.729	0.76	5

**Table 3:** Physical performance of fillers.

Type	Hydrophilic Coefficient	Density (g/cm^3^)	Specific Surface Area (m^2^/g)	Percent Pass 0.075 mm (%)
Fe_3_O_4_ powder	1.39	5.18	0.5	99
Magnetite powder	1.23	4.72	0.32	99
Limestone powder	0.63	2.75	0.78	92.1

**Table 4:** Test scheme and optimum asphalt content.

Types	CFe Replacement Rate (%)	Limestone Powder Content (%)	Aggregate	Optimum Asphalt Content (%)
Control group	0	100	0.075–16 mm limestone	4.8
CFe0	0	100	0.075–2.36 mm limestone + 2.36–4.75 mm magnetite + 4.75–16 mm limestone	4.7
CFe25	25	75		4.7
CFe50	50	50		4.6
CFe75	75	25		4.5
CFe100	100	0		4.4

**Table 5:** The heating rate within 0–2 min and the heating time to reach 0 °C.

Type	CFe100 (−5 °C)	CFe50 (−5 °C)	CFe100 (−10 °C)	CFe50 (−10 °C)	CFe100 (−15 °C)	CFe50 (−15 °C)
Heating rate K (°C/min)	8.35	8.13	6.37	6.05	5.38	5.20
Heating time to reach 0 °C (s)	43.8	46.7	102.7	106.4	147.5	151.4

## Data Availability

Not applicable.
